# Childhood leukaemia and socioeconomic status

**DOI:** 10.1038/bjc.2012.170

**Published:** 2012-06-12

**Authors:** S Franceschi

**Affiliations:** 1 International Agency for Research on Cancer, 150 cours Albert Thomas, 69372 Lyon cedex 08, France

**Sir,**

Kroll *et al* (2011) report a significant relationship over an extended period (1976–2005) between childhood lymphoid leukaemia in Britain and a measure of affluence, incidence declining across five levels of increasing relative deprivation. Among several potential explanations for their finding, the authors incline towards the disturbing possibility of uneven investigation of ill children by British paediatricians, mentioning in support only that ‘health service inequalities undoubtedly persist in modern Britain’. Many factors are related to the deprivation including family size, therefore, adjusting for any known to influence childhood leukaemia risk is clearly relevant. In a notable study by the Childhood Cancer Research Group, Dockerty *et al* (2001) found that adjusting for maternal parity and maternal age removed statistical significance from the relationship with deprivation shown in their data. It would be of great interest if the effects of such adjustment on the relation with deprivation could be reported.
